# 

**Published:** 1906-02

**Authors:** 


### CONTENTS.

ORIGINAL COMMUNICATIONS—

SOME OF THE USES AND ABUSES OF THE GALVANO-CAUTERY. By Arthur G. Hobbs, M.D., Atanta, Ga.... 721
GONORRHEA AND SOME OF ITS RESULTS. By E. G. Ballenger, M.D., Atlanta, Ga.... 733
BALLADS OF OLD DOCTORS.... 740

EDITORIAL NOTES AND COMMENTS—

OPTIMISM.... 741

NEWS AND NOTES.... 744

BOOK REVIEWS—

GRAY'S ANATOMY. (Gray).... 751
GREEN'S PATHOLOGY. (Green).... 752
A TEXT-BOOK ON MODERN MATERIA MEDICA AND THERAPEUTICS. (Stevens).... 752
THE MEDICAL NEWS POCKET FORMULARY. (Thornton).... 753
GALL-STONES AND THEIR SURGICAL TREATMENT. (Moynihan).... 753
ESSENTIALS OF MATERIA MEDICA, THERAPEUTICS, AND PRESCRIPTION WRITING. (Morris).... 754
DIFFERENTIAL DIAGNOSIS AND TREATMENT OF DISEASE. (Caillé).... 754
BERG'S SURGICAL DIAGNOSIS. (Berg).... 755
CLINICAL METHODS. (Hutchison).... 755

CONTENTS CONTINUED.... X

